# *Pseudomonas syringae* Pathovar *syringae* Infection Reveals Different Defense Mechanisms in Two Sweet Cherry Cultivars

**DOI:** 10.3390/plants14010087

**Published:** 2024-12-31

**Authors:** Claudia Carreras, Alan Zamorano, Luis Villalobos-González, Paula Pimentel, Lorena Pizarro, María Francisca Beltrán, Weier Cui, Manuel Pinto, Franco Figueroa, Carlos Rubilar-Hernández, Analia Llanes, Assunta Bertaccini, Nicola Fiore

**Affiliations:** 1 Facultad de Ciencias Agronómicas, Departamento de Sanidad Vegetal, Universidad de Chile, La Pintana 8820808, Chile; claudia.carreras@gmail.com (C.C.); agezac@uchile.cl (A.Z.); cuiweierpku@gmail.com (W.C.); 2 Programa de Doctorado en Ciencias Silvoagropecuaria y Veterinarias, Campus Sur, Universidad de Chile, La Pintana 8820808, Chile; 3 Centro de Estudios Avanzados en Fruticultura (CEAF), Rengo 2940000, Chile; luisvillalobosg1@gmail.com (L.V.-G.); ppimentel@ceaf.cl (P.P.); 4 Instituto de Ciencias Agroalimentarias, Animales y Ambientales, Universidad de O’Higgins, San Fernando 3070000, Chile; lorena.pizarro@uoh.cl (L.P.); manuel.pinto@uoh.cl (M.P.); franco.figueroa@uoh.cl (F.F.); carlos.rubilar@uoh.cl (C.R.-H.); 5 Centro de Biología de Sistemas para el Estudio de Comunidades Extremófilas de Relaves Mineros (SYSTEMIX), Universidad de O’Higgins, Rancagua 2820000, Chile; 6 Pinto Piga Seeds S.A., Joaquín Rodríguez 7317, Macul 7820206, Chile; fbeltran@pintopiga.com; 7 Laboratorio de Fisiología Vegetal-Interacción Planta-Ambiente, Departamento de Ciencias Naturales, Universidad Nacional de Río Cuarto, Ruta Nac. 36—Km. 601, Río Cuarto X5804BYA, Córdoba, Argentina; allanes@exa.unrc.edu.ar; 8 Department of Agricultural and Food Sciences, *Alma Mater Studiorum*—University of Bologna, 40127 Bologna, Italy; assunta.bertaccini@unibo.it

**Keywords:** plant pathogenic bacteria, transcriptome, plant–pathogen interaction

## Abstract

*Pseudomonas syringae* pv. *syringae* is the main causal agent of bacterial canker in sweet cherry in Chile, causing significant economic losses. Cultivars exhibit diverse susceptibility in the field and the molecular mechanisms underlying the differential responses remain unclear. RNA-seq analysis was performed to characterize the transcriptomic response in cultivars Santina and Bing (less and more susceptible to *P. syringae* pv. *syringae*, respectively) after 1 and 7 days post-inoculation (dpi) with the bacterium. Symptoms of bacterial canker became evident from the fifth day. At 1 dpi, cultivar Santina showed a faster response to infection and a larger number of differentially expressed genes (DEGs) than cultivar Bing. At 7 dpi, cultivar Bing almost doubled its DEGs, while cultivar Santina tended to the normal DEG levels. *P. syringae* pv. *syringae* infection downregulated the expressions of key genes of the photosynthesis process at 1 dpi in the less susceptible cultivar. The results suggest that the difference in susceptibility to *P. syringae* pv. *syringae* is linked to the timeliness of pathogen recognition, limiting the bacteria’s dispersion through modeling its cell wall, and regulation of genes encoding photosynthesis pathway. Through this study, it has been possible to progress the knowledge of relevant factors related to the susceptibility of the two studied cherry cultivars to *P. syringae* pv. *syringae*.

## 1. Introduction

Sweet cherry (*Prunus avium* L.) is one of the most important fruits consumed in the world; the main producers are Turkey, the European Union, China, USA, and Chile [1]. During the 2023–24 seasons, Chile exported 413,979 tons [2]. Bacterial canker is one of the major diseases affecting cherry associated with a complex of bacteria of the genus *Pseudomonas*; *Pseudomonas syringae* pv. *syringae* is the main causal agent in Chile. The bacterial canker causes yield losses of 10 to 40% in commercial orchards and young plants [3], representing a significant limitation for the cherry industry.

Plants trigger defense against biotic agents through immunity activated via pathogen-associated molecular patterns (PAMPs)—plant-triggered immunity (PTI) and effector-triggered immunity (ETI). The plant recognizes the pathogen through PAMPs via transmembrane pattern recognition receptors (PRRs); or it is able to detect pathogen effectors, which are secreted into the apoplastic space or cytoplasm by a type 3 secretion system (T3SS) [4,5,6]. Then, signaling cascades are activated in which mitogen-activated protein kinases (MAPKs), G-proteins, calcium, hormones, and transcription factors (TFs) interact, regulating the expression of genes, leading to several defense responses, like production of reactive oxygen species, pathogenesis-related (PR) gene expression, cell wall modification, entry of calcium into the cell, and accumulation of plant hormones like salicylic and jasmonic acids [4,5,6,7,8]. This transcriptional reprogramming and modification can lead to susceptibility or resistance to the pathogen.

Numerous studies have reported differential responses that determine resistance/susceptibility to pathogens, both among different species and among cultivars of the same species [9,10,11]; and sweet cherry cultivars are known to vary in their resistance towards bacterial canker disease [12,13]. Studies on *Arabidopsis thaliana* (L.) Heynh. and kiwi (*Actinidia deliciosa* A. Chev.) indicate that the defense mechanisms against pathogens of the *Pseudomonas* genus are focused on the stimulation of PTI, activating genes involved in metabolism and cell wall signaling [14,15,16]. Additionally, upon *P. syringae* pv. *actinidae* infection in kiwi, it was determined that most of the differentially expressed genes (DEG) were involved in the salicylic acid (SA) signaling pathway [nonexpressor of PR genes 1 (NPR1), TGACG-binding factors (TGAs), and PR1], being upregulated in the less susceptible cultivars [16]. In apricot (*Prunus armeniaca* L.), candidate genes involved in resistance to *Pseudomonas syringae* are potentially related to signal transduction through phosphoinositides (PI) and hormones such as abscisic acid (SA) and jasmonic acid (JA) [17]. When the SA pathway is activated, a defense response at the site of infection is often triggered in distal plant parts in the form of systemic acquired resistance (SAR). The detection of pathogens causes an increase in SA levels with the induction of PR genes. There are two pathways in the biosynthesis of SA, the isochorismate (IC) and the phenylalanine ammonia lyase (PAL) pathways [18,19]. The proteins that participate in SA pathways are NPR family proteins (NPR1, NPR3, NPR4), SA-binding proteins (SABPs), and many glutathione S-transferase and transcription factors like WRKY and TGA [18,19].

Evaluation of gene expression provided insights into how the cherry tree cultivars Santina and Bing respond to *P. syringae* pv. *syringae*. It was hypothesized that the differences between the two transcriptomes may reflect the differences in resistance or susceptibility to *P. syringae* pv. *syringae* observed between the two cultivars.

## 2. Results

### 2.1. Symptoms

**Figure 1:**
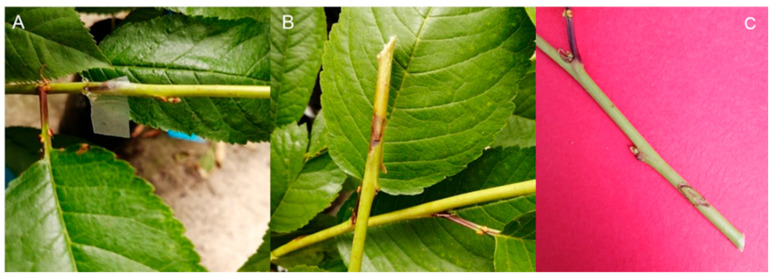
Symptoms at 7 dpi in cherry sweet trees inoculated with *Pseudomonas syringae* pv. *syringae* 11116B1 strain: (**A**) symptoms of gum secretion; (**B**) symptoms of necrosis; (**C**) mock-inoculated.

On 23 November 2022, cherry tree cultivars Santina and Bing on Gisela 12 rootstock were inoculated with *P. syringae* pv. *syringae* 11116B1 strain. At 5 dpi, gum secretion and necrosis symptoms became evident around the inoculation sites. At 7 dpi, the inoculated trees showed the presence of necrosis around the inoculation zone in 44.4% and 33.3% of the cultivars Santina and Bing trees, respectively. Additionally, 11.1% of the trees from both cultivars exhibited gum secretion (Figure 1). Mock-inoculated trees did not display symptoms of necrosis or gum secretion during the whole experiment. The presence of bacteria in plants of cultivars Santina and Bing at 1 and 7 dpi was confirmed in inoculated trees (T1 and T3) and not detected in those mock-inoculated (T2 and T4), as evidenced by de novo assembly mapping against the reference genome of *P. syringae* pv. *syringae* strain 11116B1 (GenBank accession number: GCA_029383325.1) (Supplementary Table S1). These results indicate that inoculation and establishment of the bacteria were successful in the inoculated trees.

### 2.2. Transcriptome Datasets of Sweet Cherry Tree Cultivars Bing (Susceptible) and Santina (Less Susceptible)

Of the approximately 57 million Illumina raw reads with a length of 151 nt (Supplementary Table S2) within each dataset, 94.9–97.8% reached the quality of Q20, and 88.4–94.2% of the reads reached the quality of Q30. Quality trimming generated 48,620,934 to 75,719,399 trimmed reads, corresponding to 95.8% to 98.61% of the raw reads, rendering average read lengths of 128.45 to 138.18 nt. For 24 datasets, 33% to 54.4% of the trimmed reads were mapped to the reference transcriptome of *P. avium* cultivar Satonishiki, covering 42% to 60% of the total of 35,009 transcripts.

### 2.3. Differentially Expressed Genes in Response to Pseudomonas syringae pv. syringae 11116B1 Strain

**Figure 2:**
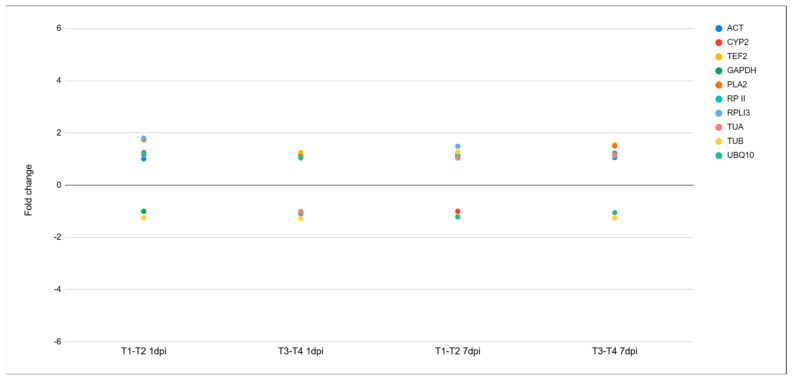
Expression of housekeeping genes at 1 and 7 dpi. T1–T2: cultivar Santina cherry trees inoculated with *Pseudomonas syringae* pv. *syringae* 11116B1 vs. mock-inoculated; T3–T4: cultivar Bing cherry trees inoculated with *P. syringae* pv. *syringae* 11116B1 vs. mock-inoculated. *FDR < 0.05 and |FC| > 2. *ACT*: actin 2/7; *CYP2*: cyclophilin; *TEF2*: translation elongation factor 2; *GAPDH*: glyceraldehyde-3-phosphate dehydrogenase; *PLA2*: phospholipase A2 beta; *RP II*: RNA polymerase subunit; *RPLI3*: 60S ribosomal protein L I 3; *TUA*: tubulin alpha-5; *TUB*: tubulin beta-1; *UBQ10*: ubiquitin 10.

The DEGs were chosen based on the criteria of FDR < 0.05 and |FC| > 2. To confirm the quality of RNA-seq, the expression of ten housekeeping genes was assessed [20]; none of these reference genes was significantly differentially expressed; suggesting that the transcriptome sequences met the quality requirements for analysis (Figure 2).

**Figure 3:**
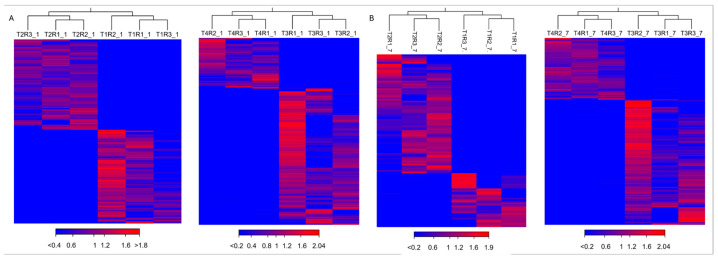
Clustered heat map of DEGs: (**A**) 1 dpi; (**B**) 7 dpi. T1: cultivar Santina cherry trees inoculated with *Pseudomonas syringae* pv. *syringae* 11116B1; T2: mock-inoculated cultivar Santina cherry trees; T3: cultivar Bing cherry trees inoculated with *P. syringae* pv. *syringae* 11116B1; T4: mock-inoculated cultivar Bing cherry trees.

The hierarchical clustering heatmap of DEGs showed that the three repeats of each treatment were highly correlated; the difference between the mock inoculated (T2 and T4) and the treatments inoculated with *P. syringae* pv. *syringae* (T1 and T3) could be distinguished at 1 and 7 dpi, indicating that the bacteria had a significant effect (Figure 3).

**Figure 4:**
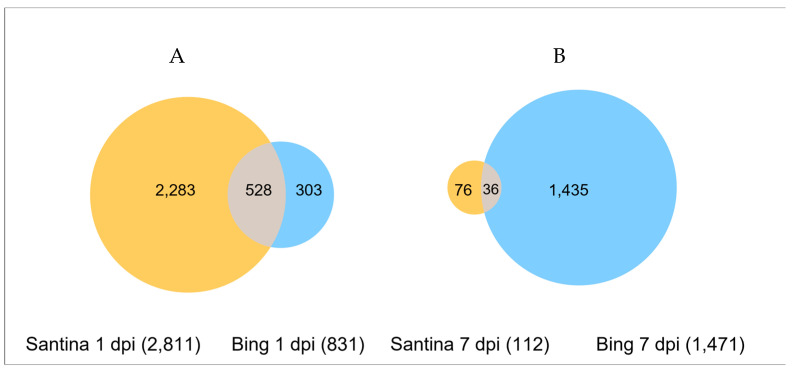
Venn diagrams showing differentially expressed sweet cherry genes post-inoculation with *Pseudomonas syringae* pv. *syringae* 11116B1 in cultivars Santina and Bing: (**A**) 1 dpi; (**B**) 7 dpi.

Different gene-expression patterns were triggered in the sweet cherry cultivars Santina and Bing. Compared with the mock-inoculated, at 1 dpi, cultivar Santina expressed a greater number of DEGs (2811) than cultivar Bing (831) (Figure 4A); while 1018 upregulated and 1265 downregulated DEGs were observed in cultivar Santina and 194 upregulated and 109 downregulated DEGs in cultivar Bing. At 7 dpi, the behavior was inverse; cultivar Bing showed more DEGs (1471) (Figure 4B), and the upregulated DEGs were 965 and 19, in cultivars Bing and Santina, respectively. This difference indicates that cultivar Santina responded earlier against *P. syringae* pv. *syringae* strain 11116B1 inoculation than cultivar Bing.

### 2.4. Functional Categories and Gene Ontology Enrichment

To understand the involvement of possible biological processes or pathways in response to *P. syringae* pv. *syringae* in the two cultivars with contrasting susceptibility, Gene Ontology (GO) was used with functional enrichment analysis by DAVID. GO classifications are divided into three categories: biological processes, cellular components, and molecular functions. At 1 dpi, in cultivar Santina 2095 (91.7%) and in cultivar Bing 285 (94.05%) of the DEGs belonged to one category. At 7 dpi, these values were 74 (97.36%) in cultivar Santina and 1341 (94.45%) in cultivar Bing.

**Figure 5:**
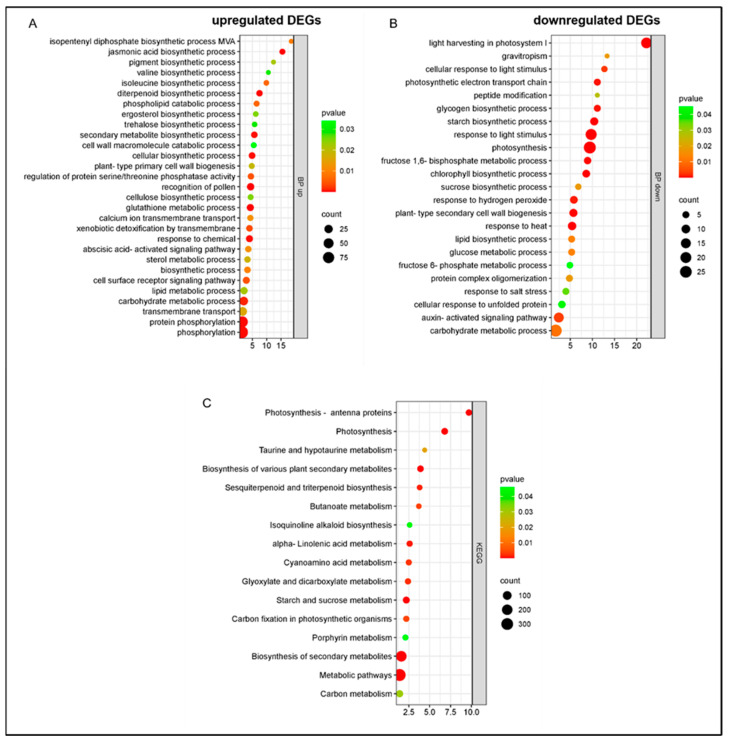
Gene Ontology enrichment of DEGs in response to *Pseudomonas syringae* pv. *syringae* inoculation in cultivar Santina at 1 dpi: (**A**) biological process, upregulated DEGs; (**B**) biological process, downregulated DEGs; (**C**) KEGG pathways. BP: biological process. Y axis: GO pathways; X axis: enrichment factors.

In cultivar Santina, at 1 dpi, the upregulated genes were significantly enriched in 29 biological processes linked to secondary metabolites and their precursors like the isopentenyl diphosphate biosynthetic process, mevalonate pathway, and precursors for terpenoids: pigment, valine, isoleucine, and diterpenoid, as well as jasmonic and abscisic hormonal processes (Figure 5A). The downregulated genes were significantly enriched in 23 biological processes mainly in relation to photosynthesis (Figure 5B), indicating a possible reallocation of resources towards the immune response. The principal enriched KEGG (Kyoto Encyclopedia of Genes and Genomes) pathways were photosynthesis–antenna proteins and photosynthesis (Figure 5C).

**Figure 6:**
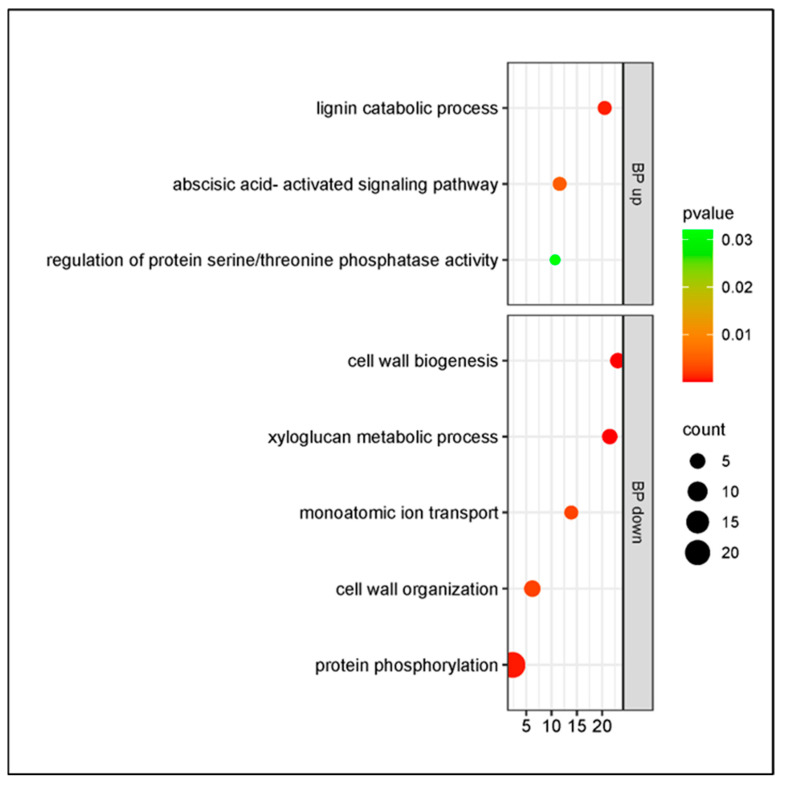
Gene Ontology enrichment of DEGs in response to *Pseudomonas syringae* pv. *syringae* inoculation in cultivar Bing at 1 dpi. BP up: biological process, upregulated DEGs; BP down: biological process, downregulated DEGs. Y axis: GO pathways; X axis: enrichment factors.

The samples from cultivar Bing revealed a smaller number of biological processes than those from cultivar Santina at 1 dpi. The upregulated genes were significantly enriched in the lignin catabolic process and abscisic acid-activated signaling pathway; the downregulated DEGs were enriched in cell wall biogenesis and xyloglucan metabolic process (Figure 6). In the xyloglucan metabolic process, the DEGs were downregulated, in conjunction with the downregulation of the enzyme xyloglucan endotransglucosylase/hydrolase (XTHs) and the upregulation of lignin catabolic genes, suggesting an effect on the strength of the cell wall. In cultivar Santina, this process was upregulated and the DEGs of secondary cell wall biogenesis were downregulated.

**Figure 7:**
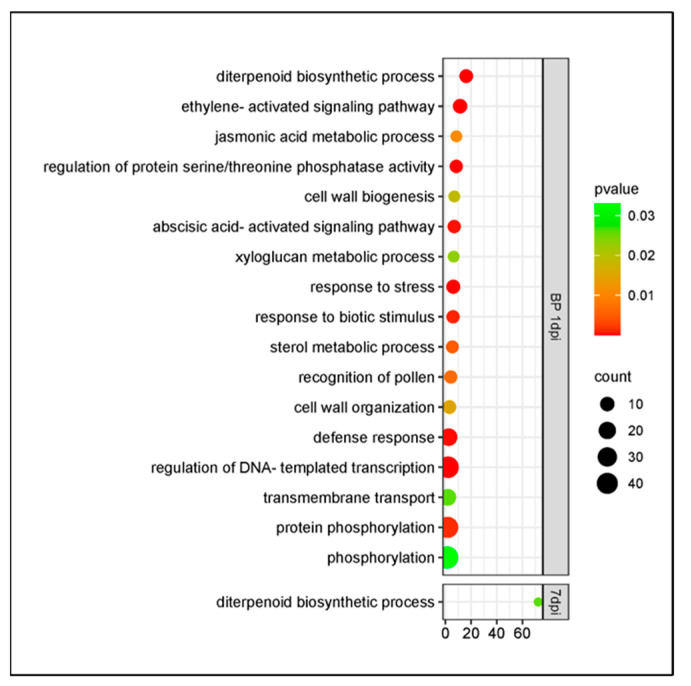
Gene Ontology enrichment of DEGs in response to *Pseudomonas syringae* pv. *syringae* inoculation shared by cultivars Santina and Bing at 1 and 7 dpi. BP: biological process. Y axis: GO pathways; X axis: enrichment factors.

The cultivars Santina and Bing shared DEGs linked to plant defenses like the diterpenoid biosynthetic process, response to stress, response to biotic stimulus, and defense response (Figure 7). Also presenting enriched jasmonic acid metabolic processes, ethylene, and abscisic acid-activated signaling pathways, these DEGs were upregulated in both cultivars. However, these cultivars showed shared DEGs in the opposite direction; cell wall biogenesis and the xyloglucan metabolic process were upregulated in cultivar Santina and downregulated in cultivar Bing. At 7 dpi, the diterpenoid biosynthetic process was upregulated in both (Figure 7).

**Figure 8:**
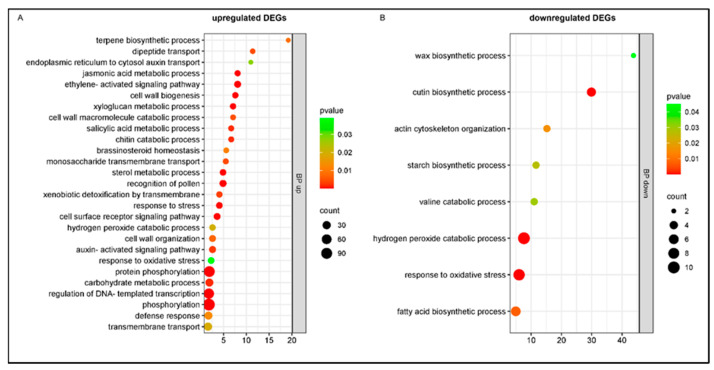
Gene Ontology enrichment of DEGs in response to *Pseudomonas syringae* pv. *syringae* inoculation in cultivar Bing at 7 dpi: (**A**) biological process; (**B**) cellular component. Y axis: GO pathways; X axis: enrichment factors.

Analyzing the upregulated genes in the cultivar Bing at 7 dpi, the biological categories overrepresented the terpene biosynthetic process, dipeptide transport, response to stress, cell surface receptor signaling pathway, hydrogen peroxide catabolic process, response to oxidative stress, and defense response involved in plant immune response. Also, genes related to plant hormone biosynthesis showed enrichment: jasmonic and salicylic acid metabolic processes, ethylene and auxin-activated signaling pathways, and brassinosteroid homeostasis; and cell wall biogenesis and organization (Figure 8A). The downregulated genes were enriched in wax and cutin biosynthetic processes (Figure 8B).

**Figure 9:**
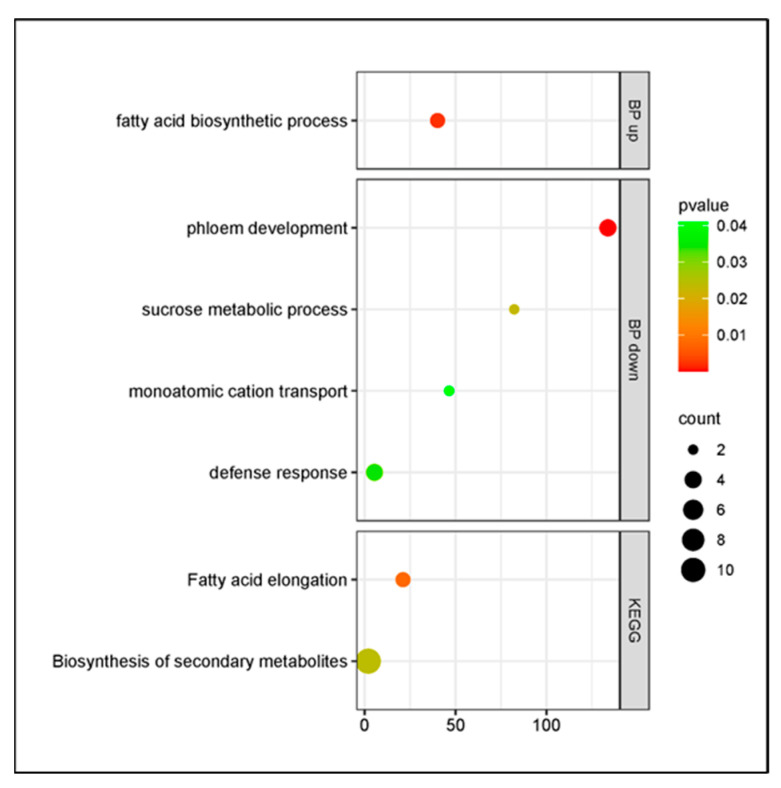
Gene Ontology enrichment of DEGs in response to *Pseudomonas syringae* pv. *syringae* inoculation in cultivar Santina at 7 dpi. BP: Biological process. Y axis: GO pathways; X axis: enrichment factors.

In the cultivar Santina, the response upon *P. syringae* pv. *syringae* inoculation was observed to decrease, although it kept active the metabolic pathway of biosynthesis of secondary metabolites and showed enriched phloem development and fatty acid biosynthesis, presenting down- and upregulated genes, respectively (Figure 9).

All the DEGs were annotated by the Gene Ontology browser QuickGO (www.ebi.ac.uk/QuickGO, accessed on 10 July 2024) and the UniProt database (www.uniprot.org, accessed on 10 July 2024). Some genes were upregulated in the cultivar Santina at 1 dpi and were not differentially expressed in Bing, like autophagy-related proteins: universal stress protein PHOS32, methyl-CpG-binding domain-containing protein 13, serine/threonine-protein kinase BLUS1, probable inactive poly [ADP-ribose] polymerase SRO2, and ureide permease 2 (Supplementary Table S3). Other genes downregulated in cultivar Santina at 1 dpi and not differentially expressed in cultivar Bing include those encoding repetitive proline-rich cell wall protein 1, protein sieve element occlusion, and genes related to photosynthesis, like chlorophyll a-b binding protein, photosystem I subunit O, and photosystem II core complex proteins psbY.

Also, detected genes were downregulated at 1 dpi in cultivar Santina and then, at 7 dpi in cultivar Bing (Supplementary Table S3). Among these genes were encoded early light-induced protein 1, isochorismate synthase, protein NSP-interacting kinase domain, and zingipain-2. Other genes differentially regulated at 1 dpi in cultivar Santina and detected at 7 dpi in cultivar Bing were heavy metal-associated isoprenylated plant protein; a short-chain alcohol dehydrogenase/reductase gene (SDR7); WAT1-related protein, related to secondary cell wall thickness; glucan endo-1,3-beta-D-glucosidase, involved in cell wall degradation; glutamate receptor, involved in biotic stress and light-signal transduction; wall-associated receptor kinase, involved in the detection of pathogen signals; mandelonitrile lyases, also identified in *Prunus persica* cultivar GS305 (peach) upon plum pox potyvirus (PPV) infection; and cellulose synthase, which plays a critical role in cell wall biosynthesis.

The genes involved in the SA pathway were analyzed (Supplementary Table S4). In both cultivars, the SA pathway was activated in response to *P. syringae* pv. *syringae*, presented as differentially regulated at 1 dpi the genes encoding SABP2, maintaining differential expression up to 7 dpi in the cultivar Bing. The cultivar Santina showed differential expression of the follow genes at 1 dpi: MAPKs, MAPKKK5, and MAPKKK7; the cultivar Bing showed differential expression at 7 dpi for MAPK, MAPKK9, MAPKKK17 and MAPKKK18. PAD4 (phytoalexin 4 deficiency), pathogenesis-related protein PR-4, isochorismate synthase, and NDR1/HIN1 showed differential expression in cultivar Santina, NDR1/HIN1-like protein 13-3 and 6, NDR1/HIN1-like protein 1 and 6 in cultivar Bing. Differentially downregulated genes encoding NPR4, PR-1 were exclusively detected in cultivar Santina. Meanwhile, genes encoding PAL, protein SAR DEFICIENT 1 were uniquely differentially regulated in cultivar Bing at 1 dpi. At 7 dpi, no genes of the SA pathway were differentially expressed in cultivar Santina, except pathogenesis-related protein PR-4.

The expression of WRKY transcription factors was influenced by the increase in SA levels. The genes that encode WRKY were shown to be differentially expressed in both cultivars; WRKY46 was upregulated in cultivar Santina at 1 dpi, and in cultivar Bing at 7 dpi. In contrast, the genes encoding WRKY71 were downregulated at 1 dpi in cultivar Santina and 7 dpi in cultivar Bing. Some were detected only in cultivar Santina, such as WRKY (Supplementary Table S5).

The cultivar Santina showed differentially expressed WAK-associated genes (PAMP receptors) at 1 dpi, while in cultivar Bing, this occurred at 7 dpi (Supplementary Table S6). Similar behavior was observed in other PAMP-recognition receptors, LRRs; at 1 dpi, cultivar Santina showed a greater number of these genes compared with cultivar Bing (25 vs. 2); however, at 7 dpi, these genes were not differentially expressed in cultivar Santina, while cultivar Bing exhibited 14 genes encoding LRRs.

To validate the RNA-seq results, ten genes were selected, which showed different patterns of expression in both cultivars Santina and Bing. Their expression levels were analyzed by RT-qPCR using gene-specific primers (Supplementary Table S7). The qPCR results and RNA-seq levels were statistically significant (r = 0.63 *p*-value = 4.5 × 10^−4^).

## 3. Discussion

*P. syringae* pv. *syringae* is one of the causal agent of bacterial canker in sweet cherry trees, the principal symptoms are gummosis, cankers in the branches and trunk, death of branches, and total tree collapse, generating significant yield losses [21]. During this study, the *P. syringae* pv. *syringae*-inoculated trees showed gummosis and necrosis from 5 dpi, while the mock-inoculated did not exhibit symptoms throughout the trial. Additionally, no transcripts of *P. syringae* pv. *syringae* were detected in the mock-inoculated samples of either cultivar, while transcripts were found in all the plants inoculated with the bacteria (T1 and T3).

The transcriptomic analysis revealed a wide range of responses upon *P. syringae* pv. *syringae* infection. The cultivar Santina showed 29.5% more DEG at 1 dpi than the cultivar Bing; however, at 7 dpi, when the plants showed symptoms, it revealed 92.4% fewer DEGs than cultivar Bing. These results suggest that cultivar Santina can react earlier to the bacterium infection, making rapid changes with a quicker defense response. Other authors have reported comparable results when comparing susceptible and resistant cultivars [16,22,23,24,25,26]. In wild banana, *Musa acuminata* Colla, resistant to *Fusarium oxysporum* f. sp. *cubense*, the tropical race 4 pathogen triggered a higher number of DEGs during the onset of the infection [22]. Similar behavior was observed in kiwi when comparing two cultivars with contrasting susceptibility to *P. syringae* pv. *actinidiae*; the number of DEGs in resistant cultivar *Actinidia eriantha* Bentham cultivar Huate was significantly higher at 12 h post-inoculation (hpi) [16].

After 7 dpi, the cultivar Santina showed a decrease in differentially expressed genes, while the cultivar Bing behaved differently, showing an increase in total DEGs from 831 to 1471. This was also observed in other research when susceptible and resistant cultivars were compared. The resistant line of *Lupinus angustifolius* revealed massive transcriptomic reprogramming at 6 hpi, and then, only a few genes remained significantly altered; meanwhile, the susceptible cultivar showed a few DEGs at 6 hpi, and then, expression of DEGs peaked at 48 hpi, indicating the presence of a relatively delayed defense response [27].

The cultivars Bing and Santina exhibited a differential response upon *P. syringae* pv. *syringae* inoculation. At 1 dpi, toregulated genes in both cultivars were associated with secondary metabolite biosynthesis, which plays a role in the plant’s immune response against pathogens. The secondary metabolites were more present under stress conditions, acting as signal molecules to increase the expression of defense genes [28]. Meanwhile, the analysis of downregulated DEGs revealed that photosynthesis was the principal process upregulated in the cultivar Santina. The downregulation of genes related to photosynthesis has also been observed in studies comparing stress responses between resistant and susceptible cultivars. This regulation is associated with an active plant response to reduce carbon availability and limit the growth of pathogens or to prioritize the establishment of defenses over other physiological processes [27,29,30,31,32]. In *L. angustifolius*, resistance to anthracnose was associated with differential expression of “GO:0015979 photosynthesis” [27]; likewise, infection by a virulent strain of *P. syringae* in *A. thaliana* was associated with downregulation of photosynthetic genes, while the avirulent strain did not modify these genes [30]. This behavior was observed in sweet cherry cultivar Lapins at 40 dpi in response to a moderately virulent *P. syringae* pv. *syringae* strain (A1M3) [33]. Additionally, studies have revealed that PAMP recognition in plants induces suppression of nuclear-encoded chloroplast-targeted protein genes at early time points [34,35]. Chlorophyll a/b-binding protein genes were exclusively downregulated in the cultivar Santina, similar to findings in *Nicotiana benthamiana* [36], in which NbLHCB3 gene was downregulated in plants infected with turnip mosaic virus (TuMV); when it was silenced by ROS accumulation and the systemic infection of TuMV was inhibited, the transgenic plants overexpressing *NbLHCB3* gene were more susceptible to TuMV. These results suggest that it could contribute to enhancing the response against pathogens.

An additional related photosynthesis gene is the one that encodes the calcium sensing receptor (CAS), which is a specific protein spatially located on the thylakoid membrane that regulates intracellular Ca^2+^ responses by sensing changes in extracellular Ca^2+^ concentration. Playing a critical role in connecting chloroplasts to cytoplasmic–nuclear immune responses triggered during both PTI and ETI, it is also involved in the regulation of SA biosynthesis [37]. The gene expression of CAS is related to the perception of PAMPs like flg22. Upon sensing flg22, the plant upregulates CAS, which is essential for downregulation of nuclear-encoded photosynthesis-related genes and upregulation of defense gene expression through ROS-mediated retrograde signaling to the nucleus [38,39,40]. CAS was found to be differentially regulated only in the cultivar Santina at 1 dpi. This finding confirms that this cultivar may have an early response to *P. syringae* pv. *syringae*, involving CAS. Its subsequent downregulation can be explained by the homeostasis process carried out by the plant to maintain balance, thereby avoiding the overproduction of ROS and cellular damage. This also occurs in relation to the concentrations of calcium, SA, and ROS [38,39]. The decrease of these otherwise highly stable transcripts suggests that photosynthesis-related transcripts are actively degraded during ETI via mRNA decay mechanisms [41], demonstrating that the chloroplast plays an early role in integrating pathogen and defense signals [34,35,42].

The cell wall is the first physical barrier to defend against pathogens and is also involved in sensing external stresses [43]. In the cultivar Santina, the genes encoding pectin acetylesterase (PAE) showed downregulation; by contrast, in the cultivar Bing, they were upregulated. PAE is involved in enzymatic deacetylation of pectin and the functional integrity of plant cell walls; in *Arabidopsis* mutant, transgenic citrus plants and apple rootstock, overexpression of PAE decreased the tolerance to pathogens [44,45,46]. XTHs (xyloglucan endotransglucosylase/hydrolases) are one of the factors responsible for cell wall plasticity and have been associated with roles in plant resistance to pathogens [8,47,48,49,50]. At 1 dpi, the cultivar Santina showed differentially expressed (up- and downregulated) genes encoding XTHs; however, the cultivar Bing exhibited all of these downregulated, while later, at 7 dpi, it showed most of these genes upregulated. These results agree with other studies, such as those in *Capsicum annuum* L. where a rapid induction of XTH expression occurred in a pepper cultivar resistant against *Ralstonia solanacearum* compared with a susceptible cultivar. This induction was associated with the restructuring and reinforcement of the cell wall and/or the formation of tyloses and gums in xylem vessels, resulting in limitation of bacterial movement [8]. Similar, in *Pyrus pyrifolia* (Burm.f.) Nakai, a reduction of genes encoding XTH protein was found only in the cultivar susceptible to *Valsa pyri* [51]. Also, the xyloglucan metabolic process was enriched at 40 dpi in the sweet cherry cultivar Lapins upon *P. syringae* pv. *syringae* infection [33]. The results presented here suggest that cultivar Santina may be less susceptible to *P. syringae* pv. *syringae* due to the limited dispersion of the bacteria through re-modeling its cell wall.

One interesting group of genes was encoding the cell surface-localized pattern-recognition receptors (PRRs). The PRRs can be classified into different subfamilies including wall-associated receptor-like kinases (WAKs) and WAK-like kinases (WAKLs), leucine-rich repeat (LRR) RLKs and RLPs, lysine motif (LysM) receptors, and lectin-type RLKs [52,53,54]. WAK genes are inducible by SA and wounding; it has been proposed that TaWAK2 modulates pectin methyl esterase 1 (PME1) negatively in wheat, causing a more rigid cell wall, limiting the penetration and spread of *Fusarium graminearum* [54]; in tomatoes, WAK1 interacts with flagellin, inducing callose deposition and minimizing infection by *P. syringae* [55,56]. The results presented here indicate similar behavior; the cultivar Santina acted earlier than the cultivar Bing, with more susceptibility to *P. syringae* pv. *syringae*, expressing genes encoding WAK at 1 dpi that were detected at 7 dpi in cultivar Bing; additionally, more genes encoding leucine-rich repeat receptor-like protein kinases (LRR-RLKs) were upregulated at 1 dpi in cultivar Santina than in cultivar Bing.

Pathogenesis-related (PR) proteins are involved in innate immunity and play a significant role against pathogens, being able to degrade the pathogen cell walls and inhibit their growth. Overexpression of PgPR10-1 in *Arabidopsis* showed enhanced resistance against *P. syringe*, *F. oxysporum*, and *Botrytis cinerea*; similarly, it was identified as upregulated in the resistant soybean upon *Phytophthora sojae* treated with SA; this allowed speculation that it might play a key role in soybean plants’ resistance, mainly depending on SA signaling [57,58]. In this study, the results were consistent with the upregulation of the genes encoding PR-10 (major allergen Pru av 1) in the less susceptible cultivar Santina at 1 dpi, and at 7 dpi in the cultivar Bing. Also, it was found that induced PR-4, which has anti-fungal, anti-bacterial, RNase and DNase activities [59], showed upregulation in cultivar Santina at 1 dpi and in both cultivars at 7 dpi.

SA is important for plant defense against biotrophic pathogens like *P. syringae* pv. *syringae*. It is synthesized through the IC and PAL pathways, both of which utilize chorismate, the final product of the shikimate pathway. SA biosynthesis is triggered during PTI and ETI, following recognition of PAMPs and effectors. The downregulated genes encoding isochorismate synthase (ICS) were differentially expressed at 1 dpi only in cultivar Santina and in cultivar Bing at 7 dpi; the decrease in their expression can be explained by the SA homeostasis that plants must preserve [60]. Thus, the plants first detect the PAMPs and during the initial hours respond by increasing the SA concentration. However, maintaining high SA levels can be toxic to the plant, so the expression of genes related to its synthesis could inhibit or reduce their expression. The expression pattern of the SA-related DEG *NPR1* was examined, since it is a key player in SA signaling; isochorismate synthase 1 (ICS1) is a key enzyme for SA biosynthesis; NPR3/NPR4 are transcriptional repressors of SA-responsive gene expression in the absence of pathogen infection [61]; and the pathogenesis-related protein PR-1 is salicylic acid (SA)-responsive. *NPR1* was not differentially expressed in this study, while PR-1 was exclusively expressed in the cultivar Santina, in which it was downregulated at 1 dpi. NPR4 is required for basal defense against pathogens and may be involved in cross-talk between SA- and JA-dependent signaling pathways; SA binding to NPR3/NPR4 inhibits their transcriptional repressor activity, leading to derepression of their target genes and defense activation [61]; one day after inoculation, less susceptible plants down-expressed genes encoding NPR4. The genes of encoded NDR1 (non-race-specific disease resistance-1), an activator of PTI and ETI that is a component of SA-mediated resistance signaling, has been shown upregulated in the cultivar Santina at 1 dpi and at 7 dpi in cultivar Bing. It has been observed that *ndr1-1* mutants present a defective SAR response; moreover, *NDR1* has been detected upregulated during hypersensitive response (HR) in different genotypes of *Coffea arabica* L. and in the sweet cherry trees cultivar Lapins in response to *P. syringae* pv. *syringae* 11116B1 infection [33,62,63]. It was observed that the PAL and ICS pathways were induced in response to *P. syringae* pv. *syringae*. At 1 dpi, there was upregulation of *PAL* genes in the cultivar Bing, and the isochorismate synthase was downregulated at 1 dpi in the cultivar Santina and 7 dpi in cultivar Bing. These results show that the SA pathways were activated in both cultivars; however, the difference in DEGs can be interpreted as earlier initiation of this pathway in the cultivar Santina.

Analyses of the WRKY transcription factors, which are linked to the SA pathway and are one of the largest family of transcriptional regulators in plants, were also performed. It was found that *WRKY46* gene overexpression in *A. thaliana* was associated with greater resistance to *P. syringae*, and it has been established that *WRKY46*, *WRKY70*, and *WRKY53* genes can function cooperatively as positive regulators in the basal defense against *P. syringae* [64]. In cultivar Santina, *WRKY46* was overexpressed at 1 dpi, while in cultivar Bing, this occurred at 7 dpi; in addition, the cultivar Bing showed *WRKY70* upregulated at 1 and 7 dpi, while *WRKY53* was overexpressed at 7 dpi.

The genes exclusively upregulated in cultivar Santina at 1 dpi included genes involved in detoxification, such as glutathione S-transferase (GST), a group of ubiquitous and multifunctional enzymes encoded by large gene families. The roles of GST include detoxification by conjugation of toxic substances with glutathione, attenuation of oxidative stress, and participation in hormone transport; some GST genes are specifically upregulated by microbial infections [65]. Upregulated autophagy-related proteins have also been observed. Autophagy is a highly conserved mechanism in yeast, animals, and plants, which is responsible for the degradation of cytoplasmic proteins, molecules, and organelles; when plants are subjected to biotic and abiotic stresses, autophagy is activated to help cells to survive under stress conditions [66]. The short-chain alcohol dehydrogenase/reductase SDR7 is involved in cell death and defense responses regulation in rice [67]. In this study, *SDR7* was overexpressed in cultivar Santina at 1 dpi and in cultivar Bing at 7 dpi, thereby showing a differential time response. These results are consistent with those reported in rootstock Marianna 2624 after *P. syringae* pv. *syringae* inoculation [46]; Lienqueo et al. [68] suggest that *SDR7* overexpression may promote better performance of this rootstock upon *P. syringae* pv. *syringae* infection.

To further understand the response of sweet cherry trees to bacterial canker, the results of the current study are being analyzed in conjunction with those obtained in cultivar Lapin [33].

## 4. Materials and Methods

### 4.1. Plant Material and Growth Conditions

The sweet cherry cultivars Bing and Santina, respectively more and less susceptible to *P. syringae* pv. *syringae* according to field observations [13], were grafted on cultivar ‘Gisela 12’ (*P. cerasus* x *P. canescens*) and used in this study. One-year-old trees were purchased at a nursery (34°28′37″ S 70°58′44″ W). Twenty-four trees, twelve of each cultivar with the same growth conditions were randomly divided into two groups; six trees were inoculated with *P. syringae* pv. *syringae* and six trees were mock-inoculated. The trees were kept in the greenhouse at 25 °C to 27 °C and the humidity was maintained at 57–67%, under drip irrigation (2 L h^−1^) at the “Centro de Estudios Avanzados en Fruticultura” (CEAF, 34°19′21″ S; 70°50′02″ W). Sweet cherry cultivars were confirmed through a genetic analysis based on microsatellite markers [69].

### 4.2. Inoculation

All the experiments were performed with the *P. syringae* pv. *syringae* strain 11116B1, which has been described as having a high virulence [33]. The bacterial suspension was prepared from pure stock stored at −80 °C in lysogeny broth (LB) medium. A portion of the culture was taken and grown on Petri dishes containing *Pseudomonas* agar F (PAF) medium supplemented with 100 μg/mL of cycloheximide, incubated at 26 °C for 16 h. At the end of this step, a single colony was transferred to a tube containing LB liquid medium and incubated at 26 °C under shaking at 150 rpm for 12 h until the suspension reached 0.1 OD at 600 nm. The inoculum at a concentration of 108 CFU mL^−1^ was prepared in sterile distilled water (SDW). Six trees of each cultivar were immediately inoculated (T1: cultivar Santina–T3 cultivar Bing), and six trees per cultivar were not inoculated but were treated with SDW (mock) (T2: cultivar Santina–T4: cultivar Bing). One twig per tree was inoculated with a wedge cut made between the 3rd to 6th internode from the apex: in total, 20 μL of the *P. syringae* pv. *syringae* suspension or SDW was introduced, and the site was covered with sterile glycerol and sealed with parafilm. Four treatments were applied: (T1) cultivar Santina *P. syringae* pv. *syringae* inoculated; (T2) cultivar Santina mock inoculated; (T3) cultivar Bing *P. syringae* pv. *syringae* inoculated and (T4) cultivar Bing mock inoculated.

Three trees were randomly sampled for each treatment, and one twig per tree was cut over the inoculum zone after 1 and 7 days post-inoculation (dpi). For each inoculated twig, the green tissue beneath the epidermis next to the wound was sampled (50–100 mg). Symptom development was monitored.

### 4.3. RNA Extraction

Total RNA was extracted from collected samples at 1 and 7 dpi. Tissues were macerated using 1 mL of lysis kit solution and frozen in liquid nitrogen. Subsequently, the total RNA extraction was performed using Spectrum™ Plant Total RNA Kit (Sigma-Aldrich, St. Louis, MO, USA). RNA integrity was determined by visualization with agarose gel electrophoresis (1%), and the concentration and purity (OD260/280) were analyzed with an Infinite^®^ 200 PRO NanoQuant (Tecan Group Ltd., Männedorf, Switzerland). Samples with a purity higher than 1.7 (A260/280) and RNA integrity number RIN > 6.7 were sequenced at Psomagen, Inc. (Rockville, MD, USA) using the Illumina platform. The RNA library was constructed using a TruSeq stranded total RNA with Ribo-Zero Plant Kit (Illumina, San Diego, CA, USA), generating paired-end reads of 151 nt.

### 4.4. Reads Analysis and Mapping to Reference Transcriptome

The predicted transcripts from the protein-coding genes of *Prunus avium* cultivar Satonishiki [70] were used as a reference to map the reads (GenBank accession number GCA_002207925.1). The reference transcriptome contained 35,009 transcripts, corresponding to 25,841 predicted protein-coding genes. The analysis of the generated sequence raw data was carried out using CLC Genomics Workbench version 23.1 (Qiagen, Hong Kong). A high quality of data was ensured by trimming the obtained raw sequences through the removal of sequence regions outside the limit threshold, with regard to their quality (0.01 error probability), ambiguity (two nucleotides), and length (minimum of 50 nucleotides per read). The trimmed reads from each sample were mapped to the reference transcriptome using the following parameters: similarity fraction = 0.95, length fraction = 0.8, insertion/deletion cost = 3, mismatch cost = 2, and unspecific match limit = 10.

### 4.5. Differential Gene Expression Analysis

The differential expression analysis was performed with the CLC Genomic Workbench software, version 23.1 (Qiagen, Hong Kong) by comparing the results of each *P. syringae* pv. *syringae*-inoculated tree against its respective mock (SDW). The samples were normalized using the trimmed mean of M values (TMM) method. The Differential Expression in Two Groups tool in CLC Genomic Workbench software was used, using multi-factorial statistics based on a negative binomial GLM for differential gene expression between experimental and control conditions. Subsequently, comparisons between the different cherry cultivars were carried out for each sampling date. A false discovery rate (FDR) of 0.05 was applied to the multiple sample testing, calculated using the Benjamini–Hochberg method [71] to reduce the risk of false positives in multiple testing scenarios. The differential expression between two sample sets was determined based on the transcripts with absolute fold change ≥ 2.0 and FDR-adjusted *p*-value ≤ 0.05.

### 4.6. Gene Ontology Analysis

The lists of differentially expressed genes were entered into Database for Annotation, Visualization, and Integrated Discovery (DAVID, https://david.ncifcrf.gov/home.jsp, accessed on 29 July 2024) for Gene Ontology annotation, with EASE scoring (Enrichment Analysis of Superset Entities) ≤0.05 [72]. The enrichment bubble plots were generated via the SRplot web server (http://www.bioinformatics.com.cn/srplot, accessed on 29 July 2024). As described in the Results and Discussion, additional functional information about particular DEGs was inferred from annotations using the Gene Ontology browser QuickGO (www.ebi.ac.uk/QuickGO, accessed on 10 July 2024) and UniProt database (www.uniprot.org, accessed on 10 July 2024).

### 4.7. Validation of DEGs

Ten DEGs were selected based on the significance of expression changes between mock and inoculated samples (Supplementary Table S8). Primers were designed using the online software program Primer 3 (version 4.1.0; https://primer3.ut.ee/, accessed on 1 December 2023). The qPCR was carried out using Brilliant II SYBR Green QPCR Master Mix (Agilent Technologies, CA, USA) with a StepOne Real-Time PCR System (Applied Biosystems, MA, USA) following the manufacturer’s instructions. Briefly, 12 ng of cDNA and 500 nM of each primer were used in a 15 µL system, and each reaction was performed in triplicate. Relative quantification was conducted using the 2^−ΔΔCT^ value with primer efficiency correction [73]. The gene RPII (RNA polymerase subunit) was used as reference (Supplementary Table S8). Correlations between qPCR results and RNA-seq were made using Spearman’s tests and are presented as *p*-values and “r” coefficients. The images were processed using high-end scaler software [74].

## 5. Conclusions

In conclusion, this research sheds light on aspects of the differential responses between two cherry sweet cultivars against *P. syringae* pv. *syringae*. The cherry cultivars Bing and Santina responses upon *P. syringae* pv. *syringae* inoculation activated different defense mechanisms. The cultivar Santina was faster in activating its defense than the cultivar Bing, with higher expression of pathogen-responsive genes. The performance of cultivar Santina’ in response to *P. syringae* pv. *syringae* involved a higher number of genes. The possible mechanism of resistance is related to the early perception of the bacteria presence and the regulation of its dissemination from processes involving cell wall restructuring and detoxification. The selection of cultivars with higher expression of PR-10, *NDR1*, *WRKY46*, and *SDR7* or downward expression of PAE could be applied for genetic improvement. Meanwhile, the activation of autophagy-related proteins in the ‘Santina’ cultivar, such as PHOS32, BLUS1, and SRO2, suggests that cellular clearance and repair mechanisms may play an important role in defense against bacterial infections.

Control measures available for bacterial canker are limited, and effective control is almost unattainable. Its control is based mainly on preventive measures; therefore, understanding the molecular mechanisms involved in plant resistance toward *P. syringae* pv. *syringae* is an important source of knowledge for integrated management strategies and the development of new tools to enable the activation of key defense pathways in plants, like cell wall modification and *WAK* and *LRR* genes, as these genes are essential for the initiation of defense responses.

## Data Availability

Data are available from the authors upon request. The authors are writing another paper, once it is published, the data will be publicly available.

## Supplementary Materials

The following supporting information can be downloaded at https://www.mdpi.com/article/10.3390/plants14010087/s1, Table S1: Mapping analysis against the Pss11116B1 reference transcriptome; Table S2: Transcriptome Illumina sequencing results; Table S3: DEGs between cultivar ‘Bing’ and ‘Santina’ at 1–7 dpi; Table S4: Genes involved in the SA pathway; Table S5: Expression of WRKY transcription factors; Table S6: Expression of WAK-associated genes; Table S7: DNA quantification in *Prunus avium* samples. Table S8: Primers used for the validation of DEGs by qRT-PCR.
